# Macrophage Differentiation and Polarization Regulate the Release of the Immune Checkpoint Protein V-Domain Ig Suppressor of T Cell Activation

**DOI:** 10.3389/fimmu.2022.837097

**Published:** 2022-05-11

**Authors:** Gaetan Aime Noubissi Nzeteu, Stephanie Schlichtner, Sulamith David, Aylin Ruppenstein, Elizaveta Fasler-Kan, Ulrike Raap, Vadim V. Sumbayev, Bernhard F. Gibbs, N. Helge Meyer

**Affiliations:** ^1^ Division of General and Visceral Surgery, Department of Human Medicine, University of Oldenburg, Oldenburg, Germany; ^2^ Division of Experimental Allergy and Immunodermatology, Department of Human Medicine, University of Oldenburg, Oldenburg, Germany; ^3^ Medway School of Pharmacy, Universities of Kent and Greenwich, Chatham Maritime, United Kingdom; ^4^ Department of Pediatric Surgery, Children’s Hospital, Inselspital Bern, University of Bern, Bern, Switzerland; ^5^ Department of Biomedicine, University of Basel and University Hospital Basel, Basel, Switzerland; ^6^ University Clinic of Dermatology and Allergy, University of Oldenburg, Klinikum Oldenburg AöR, Oldenburg, Germany

**Keywords:** VISTA, immune checkpoint regulators, macrophage polarization, soluble immune checkpoint proteins, toll-like-receptor 4, antigen-presenting cells, T cell regulation

## Abstract

Recently, the V-domain immunoglobulin suppressor of T-cell activation (VISTA) was identified as a negative immune checkpoint regulator (NCR) that is mainly expressed in hematopoietic cells. Preclinical studies have shown that VISTA blockade results in impeded tumor growth and improved survival. Nevertheless, little is known about the physiological role of VISTA expression in macrophages. This study focused on the differential expression of VISTA in human monocytes and macrophages in order to elucidate a putative role of VISTA regulation upon macrophage polarization and activation. We observed that human peripheral monocytes constitutively release soluble VISTA, which was regulated *via* matrix metalloproteinases. However, monocyte stimulation with cytokines that induce macrophage differentiation, such as granulocyte-macrophage colony–stimulating (GM-CSF) and macrophage colony-stimulating factor (M-CSF), substantially reduced soluble VISTA release. VISTA release was further affected by various pro- and anti-inflammatory stimuli that led to macrophage polarization, where activated M1 macrophages generally released more VISTA than M2 macrophages. Additionally, we observed that stimulation of activated macrophages with the toll-like receptor 4 ligand lipopolysaccharide (LPS) led to a further decrease of soluble VISTA release. Moreover, we found that soluble VISTA impairs T cell cytotoxic activity but did not induce their programmed death. Our results suggest that VISTA is constantly produced and released in the peripheral blood where it may contribute to peripheral tolerance.

## Introduction

Macrophages play a crucial role in innate immune responses, inflammation and in antigen presentation. These cells are, however, highly heterogeneous, particularly regarding expression of surface receptors and release of pro-inflammatory and other regulatory cytokines. *In vitro*, two distinct macrophage phenotypes with differential transcriptomic profiles and functions have been intensively described. The first type, M1 macrophages, also called classically activated macrophages, are pro-inflammatory and produce cytokines such as interleukin-1β (IL-1β), IL-6, IL-12, IL-23, and tumor necrosis factor alpha (TNF-α). Conversely, the second type, namely M2 macrophages or alternatively activated macrophages, primarily release anti-inflammatory cytokines like IL-10 and the multifunctional transforming growth factor beta type 1 (TGF-β) (1). However, plasticity is a hallmark of macrophage differentiation and *in vivo* a plethora of different macrophage subtypes have been identified. Macrophage activation is favored by the presence of cytokines, where M1 macrophages are activated in the presence of IFN-γ+LPS or TNF-α, while M2 macrophages are activated by either IL-4 for the M2a macrophage subtype, Toll-like receptor (TLR) stimulation in the presence of immune complexes for M2b macrophages, IL-10 for M2c macrophages, or IL-6 for M2d macrophages. Each subtype of M2 macrophages is responsible for specific types of immunogenic function. The M2a phenotype has been mainly associated with tissue remodeling and wound healing. M2b macrophages are immunoregulative and are implicated in a Th2 response. M2c macrophages are immunosuppressive and also involved in tissue remodeling. Various tumors are often infiltrated with M2d macrophages, also called tumor-associated macrophages, that stimulate angiogenesis, tumor growth and metastasis. However, due to their high plasticity regulation of macrophage polarization is much more complex *in vivo* and different macrophage subtypes cannot always be functionally segregated. For example, both M2a and M2b macrophages might have critical roles in allergies and parasitic responses, whereas M2c are also implicated in tumor progression (2, 3). Additionally, mixed phenotypes can occur.

Macrophages, and to a lesser extent monocytes, can serve as antigen-presenting cells (APCs) and present peptide antigens to T cells *via* the major histocompatibility complex (MHC) class II. While antigen binding to the T cell receptor is the basis for a specific immune response, a second signal, which is not antigen-specific, is necessary for full activation of naive T cells. The best characterized co-stimulatory signal is the interaction of cluster of differentiation (CD) 28 on T cells with its ligands B7-1 (CD80) and B7-2 (CD86) on APCs. This second signal promotes clonal expansion, cytokine secretion, and effector function. Absence of this signal, however, facilitates T cell inactivation and induces peripheral tolerance against the respective antigen. In addition, negative signaling can directly counteract the co-stimulatory effects of CD28, thus providing negative checkpoints of T-cell activation.

Cytotoxic T lymphocyte-associated protein (CTLA-4) and Programmed Death-1 (PD-1), which both belong to the CD28/CTLA-4 subfamily of the immunoglobulin (Ig) superfamily, are key regulatory inhibitors of T cell responses. Consequently, polymorphisms of PD-1 and CTLA-4 are implicated in various autoimmune diseases (4–6). In contrast, blockade of CTLA-4 and PD-1 improves the capacity of immune cells to clear tumor cells (7). Indeed, nivolumab and ipilimumab, which respectively target PD-1 and CTLA-4, have been reported to result in improvement in cancer therapies including melanoma (8).

Besides the prototypic NCRs, CTLA-4 and PD-1, many NCRs of the B7-family and their respective ligands suppress T-cell activity and proliferation. One of these is the V domain‐containing immunoglobulin suppressor of T‐cell activation (VISTA), also called Differentiation of Embryonic Stem Cells 1 (Dies1), Gi24 and PD‐1 homolog (PD‐1H). VISTA is a 52000 to 65000 molecular weight type I immunoglobulin membrane protein with an extracellular domain which is homologous to PD‐L1 (9, 10). This protein is highly expressed on hematopoietic cells of the myeloid lineage (e.g. monocytes and macrophages). In the mouse kidney it has been shown that resident macrophages (R1) expressed constitutively high levels of VISTA, evenly in homeostatic conditions, whereas infiltrating macrophages (R2) expressed very low levels of VISTA. Furthermore, VISTA may play a major role in the repair process of ischemic injury in the kidney since VISTA dysfunction was reported to impair macrophage cytokine and chemokine production (11, 12).

A soluble form of PD-L1 has been demonstrated to be of significant importance in tumor immunotherapies (13). Moreover, degradation of cell surface protein and molecules of the extracellular matrix (ECM) is essential for different processes such as hemostasis and cell signaling. These degradations are mainly due to the actions of Matrix Metalloproteinase (MMPs). MMP-8 has been identified to play a key role in macrophage differentiation (14), whereas membrane type 1 MMP, along with VISTA, plays a role in tumor growth (15). Recently, we have shown that primary human AML cells secrete high amounts of VISTA compared to healthy mononuclear leukocytes (16). Despite many studies describing a pathological function of VISTA on APCs, little is known regarding its physiological role on resting *versus* activated macrophages, particularly also regarding soluble VISTA that can be released by proteolytic cleavage. Thus, our aim was to analyze the differential expression of VISTA in human monocytes and monocyte-derived M1 and M2 macrophages in order to elucidate the physiological role of VISTA in macrophage polarization. Furthermore, we analyzed VISTA expressions upon TLR4-mediated stimulation of M1 and M2 macrophages. Finally, we elucidated a mechanism by which soluble VISTA impairs cytotoxic activity of human T cells.

## Material and Methods

### Isolation and Cultivation of Human Monocyte-Derived Macrophages

Human PBMCs were isolated from buffy coats of healthy donors, provided by DRK Blood donation center in Springe (DRK-Blutspendedienst Niedersachsen, Germany). Isolation of PBMCs was performed by density gradient centrifugation using Histopaque-1119 (Sigma-Aldrich, Germany) as described previously (17). Monocytes were then purified using a commercial immunomagnetic isolation Kit (Miltenyi Biotec, Germany) and resuspended in RPMI 1640 media containing 25mM HEPES and 2.05mM L-glutamine (Biowest SAS, France), supplemented with 10% fetal bovine serum (FBS) – EU-approved, heat-Inactivated (Bio-Techne, USA). Cells were seeded at 1x 10^5^ monocytes per well using 96 well plates. Cells were then incubated at 37°C with 5% CO_2_ and differentiated into monocyte-derived macrophages by the addition of either 50ng/mL granulocyte macrophage-colony stimulating factor (GM-CSF, BioLegend, USA), for M1 macrophages, or 50 ng/mL macrophage-colony stimulating factor (M-CSF, BioLegend, USA), for M2 macrophages, to the culture medium. Half of the initial medium was replaced after 5 days. Macrophages were fully differentiated after 7 days and cell supernatant was replaced with fresh differentiation medium.

### Metalloproteinase Inhibition Assay

Monocytes were isolated and polarized as described in 2.1. The culture medium was supplemented with either 50 µM batimastat or 50 µM GI254023X. Fully differentiated macrophages were cultivated for another 7 days without further activation. Supernatants of monocytes differentiating into macrophages (MoM1 and MoM2) and polarized macrophages (M1 and M2) were collected for soluble VISTA analysis on days 0,1, 3, 5 and 7.

### Activation of Polarized Macrophages

Monocytes were isolated and polarized as described in 2.1. After replacement of medium on day 7 polarized macrophages were maintained in culture until day 10. Macrophages were then activated using 200 ng/ml Interferon-gamma (IFN-γ, BioLegend, USA) and 100 ng/ml lipopolysaccharide (VWR, Germany) for M1 polarization. For M2a and M2c phenotypes, macrophages were activated using 40 ng/ml Interleukin-4 (IL-4, BioLegend, USA) or 10 ng/ml Interleukin-10 (IL-10, BioLegend, USA), respectively. After 2 days of activation, cells were further incubated with or without 500 ng/ml LPS for 24 hours.

### Cell Lines

Cell lines used in this work were obtained from American Tissue Culture Collection (ATCC, USA) and were accompanied by identification test certificates.

K562 and Jurkat cells were cultured in RPMI 1640 media supplemented with 10% fetal bovine serum, penicillin (50 IU/ml), and streptomycin sulfate (50 μg/ml).

TALL-104 CD8-positive cytotoxic T lymphocytes, derived from human acute lymphoblastic leukemia (TALL), were cultured according to ATCC instructions. Briefly, ATCC-formulated Iscove’s Modified Dulbecco’s Medium was supplemented with 100 units/ml recombinant human IL-2, 2.5 μg/ml human albumin, 0.5 μg/ml D-mannitol and fetal bovine serum to a final concentration of 20% to make the complete growth medium (18).

### K562/TALL-104 and K562/Jurkat Co-Cultures

K562 cells were placed into Maxisorp 96 well plates and 100 nM PMA were added to activate protein kinase C and improve immobilization of these cell onto the plate surface. After 24 h of incubation, medium was removed and either TALL-104 cells (at a ratio of 1 K562:1 TALL-104) or Jurkat cells (at a ratio of 1 K562:1 Jurkat), present in TALL-104 or Jurkat cell culture medium, were added. Cells were co-cultured for 16 h and then separated from each other. Immobilised K562 cells were cultured in TALL-104 culture medium for 16 h as a control.

### Flow Cytometry

For flow cytometric analysis, human monocyte-derived macrophages (M1 and M2) were blocked for 30 min at 4°C with 50% FBS in autoMACS Rinsing Solution supplemented with MACS BSA Stock Solution (Miltenyi, Germany) and incubated with fluorescently labeled antibodies directed against, CD68 (Clone: REA886, Miltenyi Germany), CD80 (Clone: REA661, Miltenyi Germany), CD209 (Clone: REA617, Miltenyi Germany), and monoclonal mouse IgG2B anti-human VISTA (Clone: # 730804, R&D Systems, USA) as well as corresponding isotype control antibodies for 30 min at 4°C. Dead cells were stained with Propidium Iodide Solution (Miltenyi Biotec, Germany). The cell suspension was analyzed using a MACS Quant 16 cytometer (Miltenyi Biotec, Germany). Data were evaluated using Flowlogic 7.3 software (Inivai™ Technologies, USA).

### Immunofluorescence Microscopy

For immunofluorescence, macrophages were cultivated on cover slides (VWR, Germany). Cells were washed with PBS (Carl Roth, Germany) and fixed with methanol (VWR, Germany) for 10 min at room temperature (RT). Cover slides were then incubated for 30 min with blocking solution (PBS + 0.1% bovine serum albumin, VWR) at RT. Afterwards, cells were incubated with monoclonal mouse anti-human VISTA (R&D Systems, USA) for 1 h at RT. After washing, cells were stained with secondary goat anti-mouse IgG Alexa Fluor 594 (Thermo Scientific, USA), in the dark at RT. Cover slides were mounted with Fluoromount-G Mounting Medium, with DAPI (Thermo Scientific, USA). Image capture was performed using an Olympus BX63 (Olympus, Japan) fluorescence microscope. Images were processed by Olympus cellSense and ImageJ software.

### Cytokine and Soluble VISTA Analysis

0.3-1 ml of culture supernatant was collected during macrophage differentiation every 24 h and VISTA concentrations were quantified by ELISA. Each sample was measured in duplicate. After stimulation of macrophages, supernatants were collected in order to quantify VISTA and TNF-α concentrations by ELISA. VISTA and TNF-α levels were analyzed using a Human VISTA/B7-H5/PD-1H DuoSet ELISA, with a minimum detection of 23.4 pg/ml (R&D Systems, USA) and Human TNF-α ELISA MAX Deluxe Set, with a minimum detection 7.8 pg/ml (BioLegend, USA) according to manufacturer’s recommendations. Human IL-2 and TGF-β ELISA kits (R&D Systems, USA) were used according to manufacturer’s instructions.

### In-Cell Granzyme B Activity Assay and Detection of Cell Viability

In-cell activity of granzyme B (granzyme B catalytic activity in living cells) was analyzed by incubation of cells with 150 µM Ac-IEPD-AFC (granzyme B substrate) for 1 h at 37°C in sterile PBS as described elsewhere (16). Total cell fluorescence was then measured in living cells using excitation and emission wavelengths recommended in the Ac-IEPD-AFC manufacturer’s (Sigma) protocol. Equal numbers of cells were subjected to analysis. The activity was expressed in relative units (RU), where 1 RU is equivalent to 1 pmol of cleaved substrate per 10^6^ cells. Cell viability was analyzed using an MTS assay kit obtained from Promega (USA) according to the manufacturer’s protocol.

### Phosphoinositide-3-Kinase (PI3K) Activity

The activity of PI3K was detected as we previously reported (19). Briefly, cell lysates were first incubated with 30 µl 0.1 mg/ml substrate (PI-4,5-diphosphate) in kinase assay buffer, which was prepared from 20 mM Tris (pH 7.5), 100 mM NaCl, 0.5 mM EDTA, 8 mM MgCl2 and 40 µM ATP in a total volume of 100 µl at 37°C. Reactions were terminated by the addition of 1 ml hexane/isopropanol (13:7, v:v) and 0.2 ml 2 M KCl/HClconc (8:0.25, v:v). After vortexing the samples, organic phases were washed with HCl (0.5 ml; 0.1 M). Phosphate groups were then detected using molybdenum reagent containing 2 parts of 25 mM (NH4)6Mo7O24, 5 parts of H2SO4 (140 ml of H2SO4 conc was diluted to 900 ml with bidistilled water), 2 parts of 0.3 M ascorbic acid and 1 part of 2 mM potassium antimonyl-tartrate. The values obtained in control samples of each experiment per 1 mg protein were normalized to 100% of PI3K activity. Other values were then expressed as percentage of control (19).

### Statistical Analysis

All data are depicted as mean ± standard error of the mean (SEM). Samples were tested for normal gaussian distribution using D’Agostino-Pearson and Shapiro-Wilk normality test. As all data was normally distributed, results were analyzed using a parametric paired t-test (for two groups) and a one-way ANOVA mixed test with Holm-Sidak or Sidak post test for multiple comparisons. All calculations were performed using Prism^®^ 9.1.2 software (GraphPad Software Inc.). Statistically significance was defined as p<0.05 *, p<0.01 **, p<0.001 ***, p<0.0001 ****.

## Results

### VISTA Is Expressed in Monocytes and It’s Release Is Mediated by Metalloproteinase Cleavage

**Figure 1:**
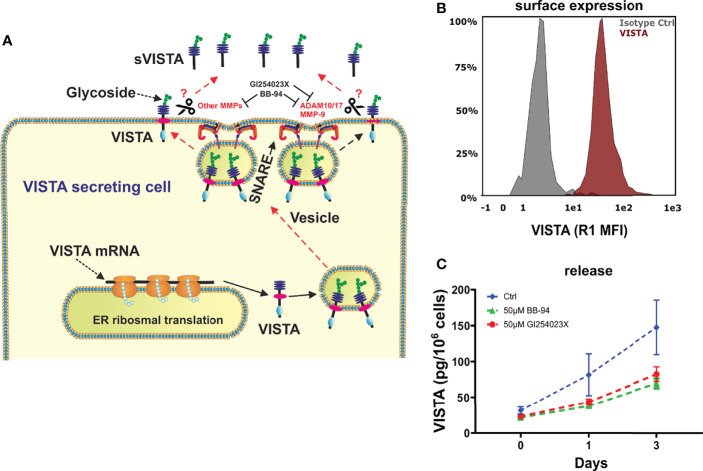
VISTA expression in human monocytes. **(A)** Scheme showing the surface expression of VISTA and proteases involved in shedding and also demonstrating the action of inhibitors. **(B)** Monocytes express VISTA on the Surface (histogram of VISTA expression on monocytes) and **(C)** release soluble VISTA *via* protease cleavage. VISTA release mediated by cleavage is reduced in the presence of matrix metalloproteinase inhibitors. Data are the mean values ± SEM (n=6).

After its expression and glycosylation, VISTA protein is transported to the cell surface. Soluble VISTA can then be generated from membrane-bound VISTA *via* proteolytic cleavage by MMPs. (**Figure 1A**) Thus, we asked whether VISTA release from monocytes is facilitated by the same mechanism. Using flow cytometry, VISTA expression was analyzed on the surface of freshly isolated monocytes (**Figure 1B**). 50 µM of batimastat, a broadband matrix metalloproteinases inhibitor (16), and 50 µM of GI254023X, an inhibitor of ADAM 10 and matrix metalloproteinase 9 (MMP-9) (20), over a period of 3 days was then used to suppress proteolytic cleavage. We observed that the presence of both metalloproteinase inhibitors strongly impaired the release of soluble VISTA into the culture medium (**Figure 1C**).

### Differentiating Monocytes But Not Fully Differentiated Macrophages Secrete Soluble VISTA

**Figure 2:**
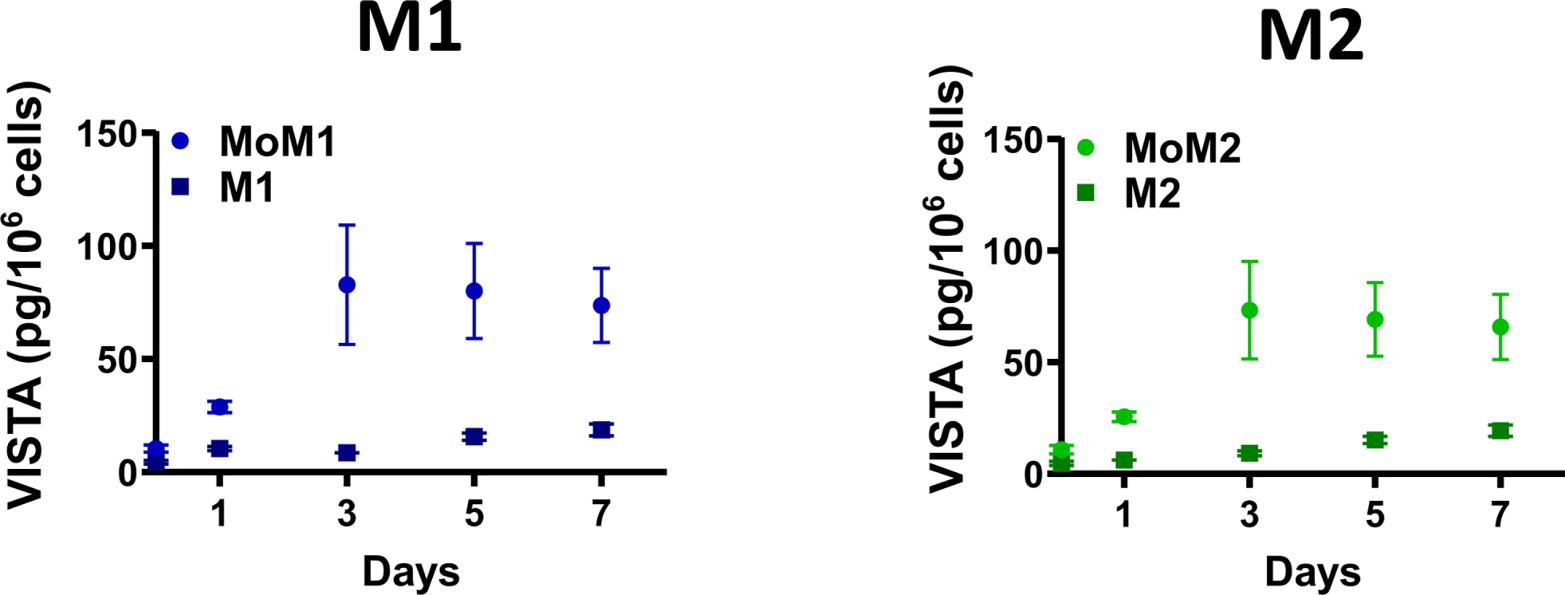
Differentiating monocytes but not fully differentiated macrophages release soluble VISTA. Monocytes were differentiated into M1 (MoM1, light-blue circles) or M2 (MoM2, light-green circles) macrophages for 7 days with GM-CSF or M-CSF, respectively. Medium was then replaced and fully differentiated M1 (dark-blue squares) and M2 (dark-green squares) macrophages were cultivated for another 7 days. VISTA concentration in culture medium was determined every second day using a VISTA sandwich ELISA. Data are the mean values ± SEM (n=3-11).

We next asked how differentiation into macrophages affects the release of soluble VISTA into the culture medium. Therefore, we determined VISTA concentrations in the supernatant of differentiating monocytes and macrophages differentiated towards an M1 or M2 phenotype by ELISA. Interestingly, monocytes differentiating into M1 (MoM1) and M2 (MoM2) released substantial amounts of soluble VISTA only in the first 3 days of culture (**Figures 2A, B**). On days five to seven soluble VISTA levels in the culture medium reached a stable plateau. Fully differentiated macrophages (M1 and M2) released significantly less soluble VISTA in comparison to MoM1 and MoM2. Successful polarization of macrophages was assessed by CD163 surface expression (**Supplementary Figure 1**)

**Figure 3:**
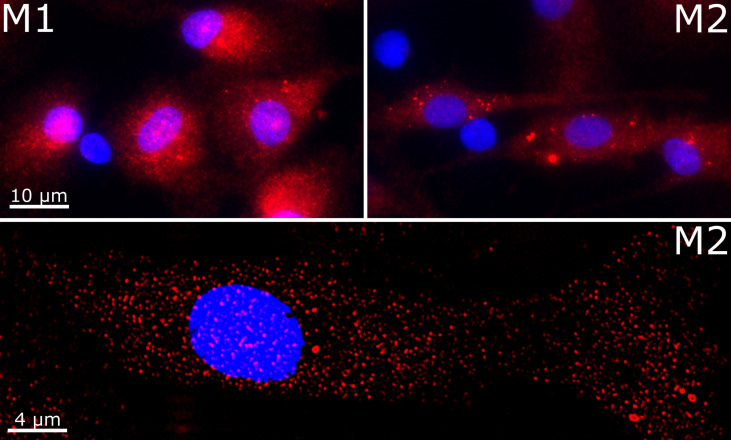
Cellular localization of VISTA in M1 and M2 macrophages. VISTA was stained in polarized macrophages with a monoclonal mouse anti-human VISTA antibody and Alexa Fluor 594-coupled secondary antibody. Lower panel shows the image deconvolution of a z-stack.

We validated the expression of VISTA in M1 and M2 macrophages using immunofluorescence microscopy. Intriguingly, we found VISTA protein was also localized intracellularly in both unstimulated M1 and M2 macrophages – most likely in intracellular vesicles (**Figure 3**).

### Effect of Different Pro- and Anti-Inflammatory Stimuli on Macrophages Regarding Surface Expression and Release of VISTA

**Figure 4:**
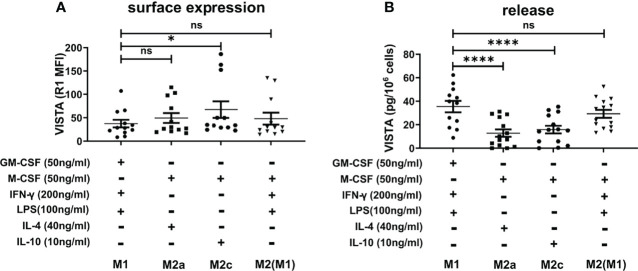
VISTA production in activated M1 and M2 macrophage subtypes **(A)** VISTA surface expression was determined by flow cytometry. **(B)** Soluble VISTA release was determined by a sandwich ELISA. Data are the mean values ± SEM (n=12-14). *p < 0.05, ****p < 0.0001, ns, non-significant (p>0.05).

**Figure 5:**
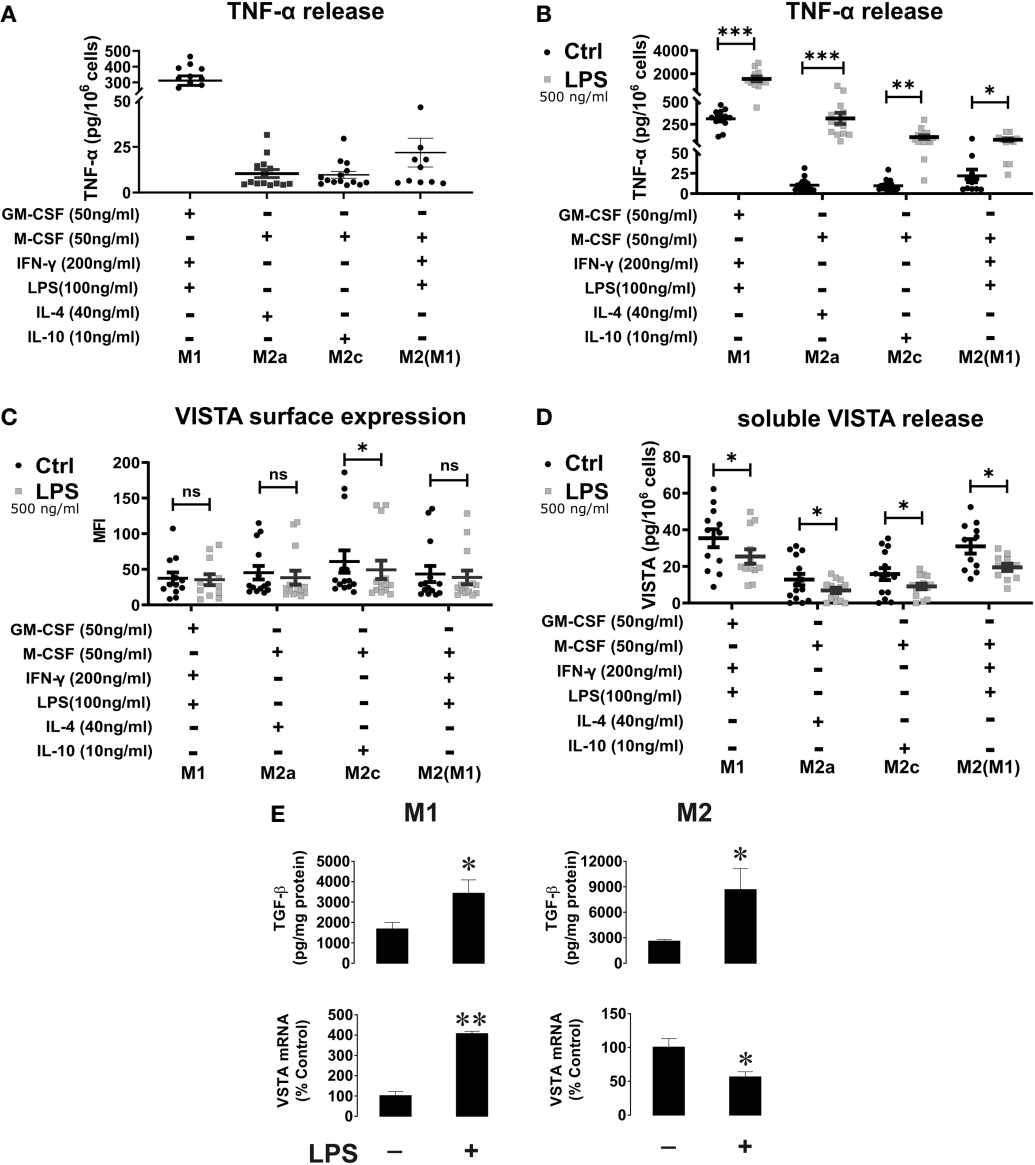
VISTA surface expression and release of soluble VISTA after LPS stimulation in macrophages. **(A)** TNF-α release from activated macrophages and **(B)** before and after stimulation with LPS for 24h. TNF-α release after stimulation with LPS confirms macrophage activation. (n=12-14) **(C)** VISTA surface expression was determined by flow cytometry before (grey squares) and after stimulation with LPS (black). **(D)** Release of soluble VISTA was determined by a sandwich ELISA. **(E)** The effects of LPS on TGF-β expression and VISTA mRNA levels in M1 and M2 macrophages. M1 and M2 macrophages were exposed to 500 ng/ml LPS for 24 h. Cell-associated TGF-β (i.e. total amount of TGF-β expressed by the cells and performing autocrine action) was measured by ELISA. VISTA mRNA levels were measured by qRT-PCR as described in *Materials and Methods*. Data are the mean values ± SEM (n=3). *p < 0.05, **p < 0.01, ***p < 0.001, ns, non-significant (p>0.05).

Macrophages play an important role in the activation and modulation of the immune system. Macrophage activation is therefore crucial for the immune response (2). Thus, we investigated whether pro- and anti-inflammatory stimuli could affect VISTA expression as well as its release from macrophages. To this end, macrophages that had been differentiated towards an M1 or M2 phenotype with GM-CSF or M-CSF, respectively, were further maintained in culture and stimulated with IFN-γ+LPS for activated M1 macrophages, IL-4 for activated M2a and IL-10 for M2c macrophages. Successful activation was assessed by CD80, CD163 and CD209 surface expression. (**Supplementary Figure 2**) Interestingly, VISTA surface expression was only slightly affected in the different M2 macrophage subtypes compared to stimulated M1 macrophages. Additionally, we stimulated M2 macrophages with IFN-γ+LPS to induce a repolarization into an M1 phenotype. Only IL-10-stimulated macrophages, which clearly represent an anti-inflammatory macrophage phenotype, show significantly elevated VISTA surface expression (**Figure 4A**). Surprisingly, VISTA release was significantly increased in M1 macrophages compared to M2a (IL-4) and M2c (IL-10) macrophages. (**Figure 4B**) Pro-inflammatory cytokine, i.e. TNF-α release from IFN-γ+LPS stimulated M2 macrophages (**Figures 5A, B**) and high surface expression of CD80 (**Supplementary Figure 2**) are indicative of a functional repolarization into an M1 phenotype. At the same time M2(IFN-γ+LPS) macrophages appear to release higher amounts of soluble VISTA compared to M2a(IL-4) and M2c(IL-10) macrophages, rather resembling the activated M1 phenotype (**Figure 4B**).

### VISTA Surface Expression and Release of Soluble VISTA After LPS Stimulation of Macrophages

Although cell surface levels of VISTA is only slightly affected by macrophage polarization, M1 macrophages released significantly more VISTA than M2 macrophages. Thus, we investigated whether further stimulation of macrophages *via* TLR-4 impacted on VISTA production. To this end, we assessed the surface expression of VISTA using flow cytometry and release of soluble VISTA by ELISA from macrophages after stimulation with high concentrations of the TLR4-ligand, LPS. We observed a tendential decrease of cell surface-expressed VISTA by flow cytometry analysis after 24h LPS stimulation (**Figure 5C**). However, only on M2c(IL-10) VISTA surface expression was significantly decreased upon LPS stimulation. Interestingly, VISTA release was reduced in all macrophage phenotypes after LPS stimulation (**Figure 5D**). At the same time soluble TNF-α was upregulated following TLR4 ligand stimulation in all phenotypes (**Figure 5B**). Importantly, we investigated whether LPS affects VISTA expression. We have recently shown that transforming growth factor beta type 1 (TGF-β) differentially regulates VISTA expression through the transcription factor Smad3 (21). We tested whether M1 and M2 macrophages produce TGF-β in response to LPS stimulation, as well as measuring VISTA mRNA levels in parallel. (**Figure 5E**) We found that the levels of cell-associated TGF-β were upregulated by LPS in both M1 and M2 macrophages. However, in M1 macrophages, both background and stimulated TGF-β levels were lower compared to those observed in M2, which is most likely related to M2 macrophage function associated with TGF-β production. However, VISTA mRNA levels were significantly upregulated in M1 macrophages after LPS stimulation whilst they were downregulated in M2 macrophages. In both types of macrophages LPS stimulation led to decrease in the release of soluble VISTA. This suggests that VISTA protein expression and secretion are controlled by differential mechanisms in both type of macrophages. The increased VISTA expression in M1 macrophages may serve as a reservoir of the protein in order to participate in opsonization of LPS-containing Gram-negative bacteria (22). Conversely, the decrease in VISTA expression in M2 macrophages is most likely to be associated with LPS-induced conversion of these cells into an M1 type which would reduce their immunosuppressive potential.

### Soluble VISTA Suppresses Activity of Tall-104 and Jurkat T Cells But Does Not Affect Their Viability

**Figure 6:**
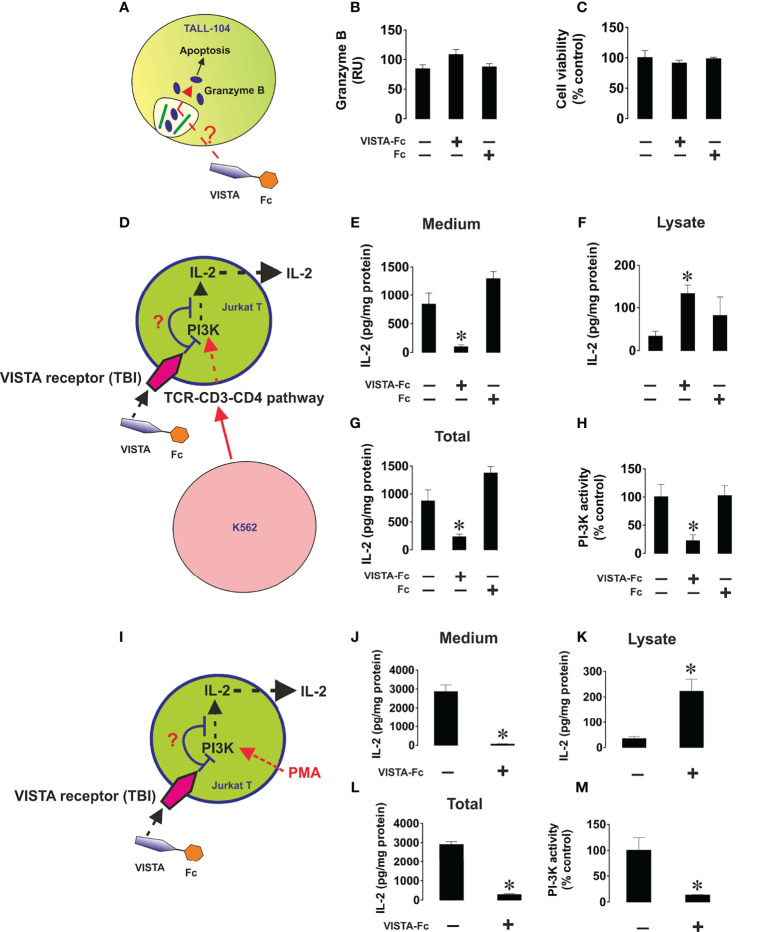
Soluble VISTA does not induce intracellular activation of granzyme B in cytotoxic T cells and prevents the release of IL-2 from helper T cells. TALL-104 cells were exposed to either 5 µg/ml VISTA-Fc or an equimolar amount of Fc for 16 h **(A)**. In-cell activity of granzyme B **(B)** and viability of TALL-104 cells were then analyzed **(C)**. K562 chronic myeloid leukemia cells, which express traces and do not release detectable amounts of VISTA or IL-2 (as confirmed by ELISA), were used as targets. K562 cells were exposed to 100 nM PMA for 24 h in 96 well Maxisorp plates for immobilization. These cells were then co-cultured with Jurkat T cells (helper T cells) at the ratio 1: 1 **(D)**. Some of the wells containing Jurkat T cells were supplied with 5 µg/ml VISTA-Fc or an equimolar amount of Fc. Cells were then co-cultured for 24 h before separation. IL-2 was measured in the medium **(E)** and cell lysates **(F)** by ELISA. Total amounts of IL-2 were also calculated **(G)**. PI3K activity was measured as outlined in Materials and Methods **(H)**. To confirm the effects observed we exposed Jurkat T cells to 100 nM PMA for 24 h in the absence or presence of VISTA-Fc **(I)**. IL-2 was then measured in the medium **(J)** and cell lysates **(K)** by ELISA. Total amounts of IL-2 were also calculated **(L)** and PI3K activity assessed **(M)**. Data are shown as mean values ± SEM for 4 independent experiments. *p < 0.05 *vs* control.

We next sought to confirm the cytotoxic T cell suppressive role which soluble VISTA was previously suggested to play. We used TALL-104 CD8-positive cytotoxic T cells and exposed them to 5 µg/ml (23, 24) VISTA-Fc protein (or an equimolar amount of Fc as a control) for 16 h followed by measurement of in-cell activity of granzyme B in living cells as well as viability of TALL-104 cells. We found that VISTA failed to induce significant upregulation of granzyme B activity in these cells and did not affect their viability (**Figures 6A–C**), which was in line with our previous observation for PMA-activated (granzyme B expressing) Jurkat T cells. (16)

**Figure 7:**
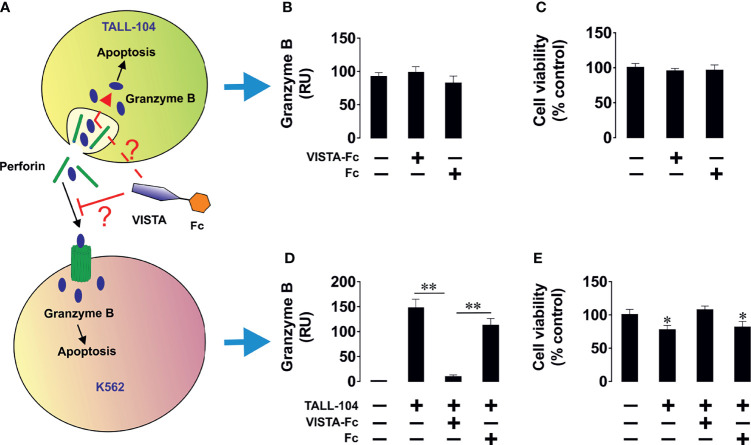
Soluble VISTA attenuates the release of granzyme B into chronic myeloid leukaemia cells by cytotoxic T cells but does not affect lymphocyte viability. K562 chronic myeloid leukemia cells, which express traces and do not release detectable amounts of galectin-9 (as confirmed by ELISA), were used as targets. K562 cells were exposed to 100 nM PMA for 24 h in 96 well Maxisorp plates for immobilization. Medium was replaced with ATCC-formulated Iscove’s Modified Dulbecco’s Medium containing 100 units/ml recombinant human IL-2; 2.5 μg/ml human albumin; 0.5 μg/ml D-mannitol and foetal bovine serum to a final concentration of 20% with or without TALL-104 cytotoxic T cells (cells in suspension). When TALL-104 cells were present, the ratio was 1 TALL-104: 1 K562 cell. Some of the wells containing TALL-104 cells were supplied with 5 µg/ml VISTA-Fc or an equimolar amount of Fc. Cells were co-cultured for 16 h before separation. In-cell activity of granzyme B and cell viability were measured in both cell types. **(A)** Scheme of the experiment; **(B)** in-cell activity of granzyme B in TALL-104 cells; **(C)** viability of TALL-104 cells; **(D)** in-cell activity of granzyme B in K562 cells; **(E)** viability of K562 cells. Data are shown as mean values ± SEM for 4 independent experiments. *p < 0.05; **p < 0.01.

Next, we wished to elucidate the effect of soluble VISTA on T helper cells. To this end we cultured CD4^+^ Jurkat T cells together with K562 cells, which will stimulate Jurkat cells *via* the T cell receptor. (**Figures 6D–H**) Alternatively, we stimulated Jurkat T cells with PMA (**Figures 6I–M**). In both cases we found that soluble VISTA inhibits the production and the release of IL-2 in Jurkat T cells most likely by downregulating PI3K activity. Finally, in order to assess whether soluble VISTA suppresses the cytotoxic activity of T cells we used K562 chronic myeloid leukemia cells, which express traces of galectin-9 and VISTA but do not release detectable amounts of these proteins. Co-cultures of K562 with TALL-140 cells were performed with or without 5 µg/ml VISTA-Fc (or an equimolar amount of Fc as control). In-cell activity of granzyme B was measured in both cell types and their viabilities were assessed. We found that VISTA (but not Fc) prevented granzyme B injection into K562 cells, thus preventing their killing (**Figure 7**). However, the presence of VISTA did not lead to granzyme B activation inside TALL-104 cells (**Figure 7**). This suggests that soluble VISTA prevents granzyme B-dependent killing of target cells by cytotoxic T cells but does not lead to programmed death of these T cells.

## Discussion

**Figure 8:**
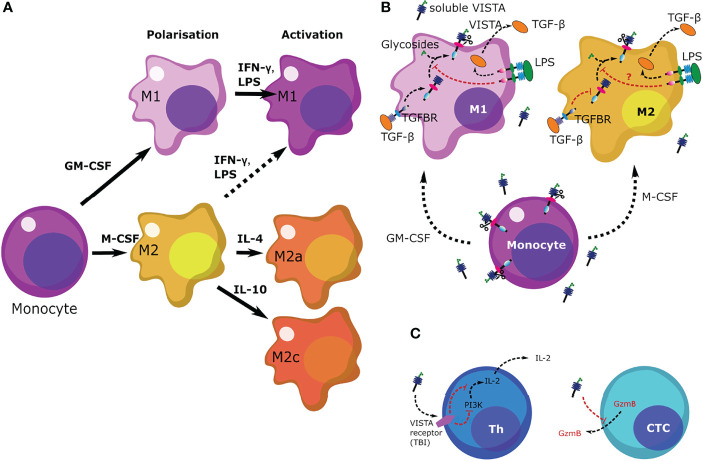
Scheme summarizing the regulation of VISTA levels in M1 and M2 macrophages as well as the role of VISTA in immune responses. **(A)** Differentiation and polarization of monocytes to macrophages in response to various stimuli. **(B)** Monocytes release higher levels of VISTA than macrophages. Upon differentiation, release of soluble VISTA is downregulated given the function of macrophages. Presence of LPS upregulates TGF-β production in both types of macrophages (M1 and M2), although M2 macrophages release more TGF-β, in accordance with their immunosuppressive role. M1 macrophages respond by upregulation of VISTA expression in response to exposure to LPS, an effect which could be triggered by TGF-β (21). However, VISTA release is downregulated. The purpose of such a response in a pathophysiological environment would trigger re-activation of the immune response in the presence of LPS. Downregulation of VISTA in this case is very likely a differential type of response to TGF-β, as reported recently. (21) **(C)** soluble VISTA acts on T cells through a receptor which remains to be identified (TBI) leading to downregulation of PI3K activity in T helper cells (Th) thus leading to decreased IL-2 production. In cytotoxic T cells (CTCs) VISTA downregulates the release of granzyme B, subsequently blocking their cytotoxic activities.

In this study we focused on the expression of VISTA in macrophages during and after polarization as well as activation with various cytokines. (**Figure 8A**) It has previously been demonstrated that VISTA is expressed as a surface membrane protein on various cell types, including monocytes (9). Here, we demonstrate that VISTA is also released from peripheral blood monocytes and, to a much lower extent, upon differentiating into macrophages. We suggest that soluble VISTA is produced by proteolytic cleavage since protease inhibitors decreased soluble VISTA release from human peripheral monocytes (**Figure 1C**). This observation is consistent with our previous finding that a truncated form of VISTA is present in the plasma of AML patients and that VISTA cleavage from the surface of THP-1 acute myeloid leukemia (AML) cells is inhibited by the protease inhibitor GI254023X (16). The release of the extracellular domain of the VISTA homolog protein PD-L1 was likewise reported to occur *via* proteolytic cleavage (25). In addition to soluble form, exosomal PD-L1 can contribute to immunosuppression *via* an anti-PD-1 response. (26) A role of exosomal immune checkpoints, including CTLA-4, PD-L1 and Tim-3 is also described in systemic immune suppression and tumor progression. (27) With this regard, it would be interesting to study the potential role of exosomal VISTA in cancer patients. Here, we showed, however, that the protease inhibitors Batimastat and GI254023X largely inhibit the release of VISTA from human monocytes of healthy donors, both to the same extent. Batimastat is an inhibitor of different matrix metalloproteinases, but not ADAM 10, while GI254023X is a specific inhibitor of ADAM 10/17 and matrix metalloproteinase 9 (MMP-9). Further studies are necessary to elucidate whether matrix metalloproteinase mediate direct release of VISTA from human monocytes, i.e. by proteolytic cleavage of its extracellular domain, or indirectly regulate its release, e.g. by modulation of exosomal VISTA.

Interestingly, we found that fully differentiated macrophages showed lower release of soluble VISTA than monocytes (**Figure 2**). Particularly, fully activated M2a and M2c macrophages show the least amount of VISTA release. We also observed that macrophages stimulated with the Toll-like-receptor 4 ligand LPS generally release less soluble VISTA, while its surface expression is hardly affected. Since VISTA release from monocytes and macrophages by proteolytic cleavage can be differentially regulated by pro- and anti-inflammatory stimuli, this suggests a potential immunomodulatory role for VISTA. We recently reported that soluble VISTA, in association with secreted galectin-9 and Tim-3, plays a role in the opsonization of gram-negative bacteria (22), indicating that VISTA crucially regulates not only adaptive immunity but also innate immune responses.

Thus far, little is known regarding the modulation of immune checkpoints following the activation of macrophage by various inflammatory and anti-inflammatory stimuli. In mice, IL-4 highly promoted the expression of PD-L2 on macrophages (28), whereas PD-L1 surface expressions were increased following stimulation with 500 ng/ml IFN-γ or after *Leishmania* infection (17, 29). In contrast, we have shown that VISTA surface expression is hardly affected upon the polarization of human macrophages into different phenotypes. Macrophage activation only promoted a slightly higher expression of VISTA in M2 *versus* M1 macrophages (**Figure 4A**). However, soluble VISTA release was variable in the different activated macrophage subtypes. Activated M1 macrophages generally released more soluble VISTA than the activated M2 macrophages subtypes, albeit moderately, with the exception of M2 stimulated with IFN-γ and LPS (**Figure 4B**). However, the increased release of TNF-α from these cells indicated a reprogramming into a pro-inflammatory M1 phenotype. In this regard, the intracellular accumulation of VISTA in M1 and M2 macrophages, might represent a dormant reservoir of VISTA protein which can rapidly be expressed on the cell surface and released into the extracellular milieu upon changing stimuli. This is in line with the observation, that VISTA is localized within the endosomal compartment in peritoneal mouse macrophages. (30) This could support the containment of acute inflammation by T-cell inactivation, employing mechanisms similar to other checkpoint molecules e.g. CTLA-4 and PD-1 as well as its ligands PD-L1 and 2. In mice CTLA-4 can be expressed in intracellular compartments and released from the cell surface according to changing conditions (31). Moreover, PD-L1 is expressed in the intracellular compartment of tumor cells and can be transported to the plasma membrane in certain settings (32). In addition, tumor cell lines and brain tumor cells showed strong constitutive expression of PDL1/2 (33). Storage of VISTA in intracellular vesicles may thus be essential for regulation of VISTA surface expression and release.

VISTA deficiency in macrophages has been previously shown to be associated with a high level of inflammatory chemokine production (11), suggesting a role in innate immune responses. Differential VISTA release from M1 and M2 macrophages indicates that VISTA may be an important immune regulator of inflammatory processes and potentially also in macrophage polarization.

Our findings on human macrophages are supported by recent investigations in mice, where macrophages resident in the CNS produced less VISTA following LPS stimulation (34). This is in contrast to a similar study with PD-L1, whose surface expression increases after LPS stimulation (35). Further studies are necessary to elucidate whether LPS stimulation over longer periods leads to a general decrease in VISTA protein production and a depletion of intracellular VISTA reservoirs in human macrophages.

Previous reports suggested that VISTA, generally downregulates T cell proliferation and cytokine production (23, 24). However, to date the role of soluble VISTA is not fully understood. It was shown that soluble VISTA-F_C_ fusion protein can inhibit T-cell proliferation and cytokine production *in vitro*, but in this case, immobilization of VISTA- F_C_ was necessary (36). In contrast, an engineered pentameric form of the extracellular domain of VISTA was shown to restrain T-cell activation *in vivo* without immobilization, suggesting a possible role of VISTA in the adaptative immune response (37).

Here, we showed that soluble VISTA can play an important role in suppression of the cytotoxic activities of T cells, by suppressing the release of granzyme B from these cells. (**Figure 8B**) Our recent findings suggested that soluble VISTA could enhance the apoptosis of cytotoxic T cells in conjunction with galectin-9 (16). Here, we confirmed that soluble VISTA on its own (in the absence of galectin-9) prevents release of granzyme B from cytotoxic T cells into target cancer cells. However, this does not lead to a significant activation of these enzyme inside cytotoxic T cells, which would otherwise induce apoptotic death. This highlights an important mechanism which granzyme B negative T cell malignancies - T cells do not normally release high amounts of galectin-9 - might use to evade immune attack by cytotoxic T cells. Since these malignant T cells normally produce VISTA, they could possibly use soluble VISTA, acting on other cytotoxic T cells, to evade immune attack. In addition, we could show that soluble VISTA suppresses the release of IL-2 from CD4+ T cells (**Figure 8C**).

## Conclusion

In this study we clearly observed that monocytes constitutively release VISTA. Secretion was markedly reduced upon GM-CSF and M-CSF induced macrophage differentiation and further regulated by macrophage activation. Additionally, we demonstrated that soluble VISTA suppresses T cell activation without inducing apoptosis. Our data support the notion that VISTA plays a role in peripheral tolerance, as has been recently demonstrated by ElTanbouly *et al.* (38). Constant release of soluble VISTA by peripheral blood monocytes into the blood plasma may result in the suppression of resting T cells. However, once monocytes are challenged with a pathogen and differentiate into macrophages, VISTA release may decrease in order to facilitate a more robust immune response. Overall, our study provides further insights into the role of soluble VISTA in innate and adaptive immune responses.

## Data Availability Statement

The raw data supporting the conclusions of this article will be made available by the authors, without undue reservation.

## Ethics Statement

Ethical review and approval was not required for the study on human participants in accordance with the local legislation and institutional requirements. The patients/participants provided their written informed consent to participate in this study.

## Author Contributions

GN, VS, BG and NM conceived the study. GN, SS, E F-K, UR, VS, BG and NM designed experiments. GN, SS, SD, AR, E F-K, VS and NM performed experiments and intepreted the data. GN, VS and NM wrote the manuscript. EF-K, UR, VS, BG and NM revised the manuscript. All authors read and approved the manuscript.

## Funding

This project was supported by Intramural Funding from the School of Medicine and Health Sciences, University of Oldenburg (FP 2017-013 to UR, BG and NM) and in part by a Swiss Batzebär Grant (to EF-K).

## Conflict of Interest

The authors declare that the research was conducted in the absence of any commercial or financial relationships that could be construed as a potential conflict of interest.

## Publisher’s Note

All claims expressed in this article are solely those of the authors and do not necessarily represent those of their affiliated organizations, or those of the publisher, the editors and the reviewers. Any product that may be evaluated in this article, or claim that may be made by its manufacturer, is not guaranteed or endorsed by the publisher.
