# Supplementary material for: Macrophage Differentiation and Polarization Regulate the Release of the Immune Checkpoint Protein V-Domain Ig Suppressor of T Cell Activation
Source: Front Immunol. 2022 May 11;13:837097. doi: 10.3389/fimmu.2022.837097 (PMC9132587; doi:10.3389/fimmu.2022.837097)
Supplement: Supplementary file 1 [file DataSheet_1.docx]

Macrophage Differentiation and Polarization Regulate the Release of the Immune Checkpoint Protein V-domain Ig Suppressor of T Cell Activation

Gaetan Aime Noubissi Nzeteu^1,2^, Stephanie Schlichtner^3^, Sulamith David^2^, Aylin Ruppenstein^2^, Elizaveta Fasler-Kan^4,5^, Ulrike Raap^2,6^, Vadim V. Sumbayev^3^, Bernhard F. Gibbs^2^, N. Helge Meyer^1,2*^

*^1^ Division of General and Visceral Surgery, Department of Human Medicine, University of Oldenburg, Oldenburg, Germany, ^2^ Division of Experimental Allergy and Immunodermatology, Department of Human Medicine, University of Oldenburg, Oldenburg, Germany, ^3^ Medway School of Pharmacy, Universities of Kent and Greenwich, Chatham Maritime, Kent, United Kingdom, ^4^ Department of Pediatric Surgery, Children’s Hospital, Inselspital Bern, University of Bern, Bern, Switzerland, ^5^ Department of Biomedicine, University of Basel and University Hospital Basel, Basel, Switzerland, ^6^ University Clinic of Dermatology and Allergy, University of Oldenburg, Klinikum Oldenburg AöR, Oldenburg, Germany*

*** Correspondence:**N. Helge Meyer
[helge.meyer@uni-oldenburg.de](mailto:helge.meyer@uni-oldenburg.de)

*
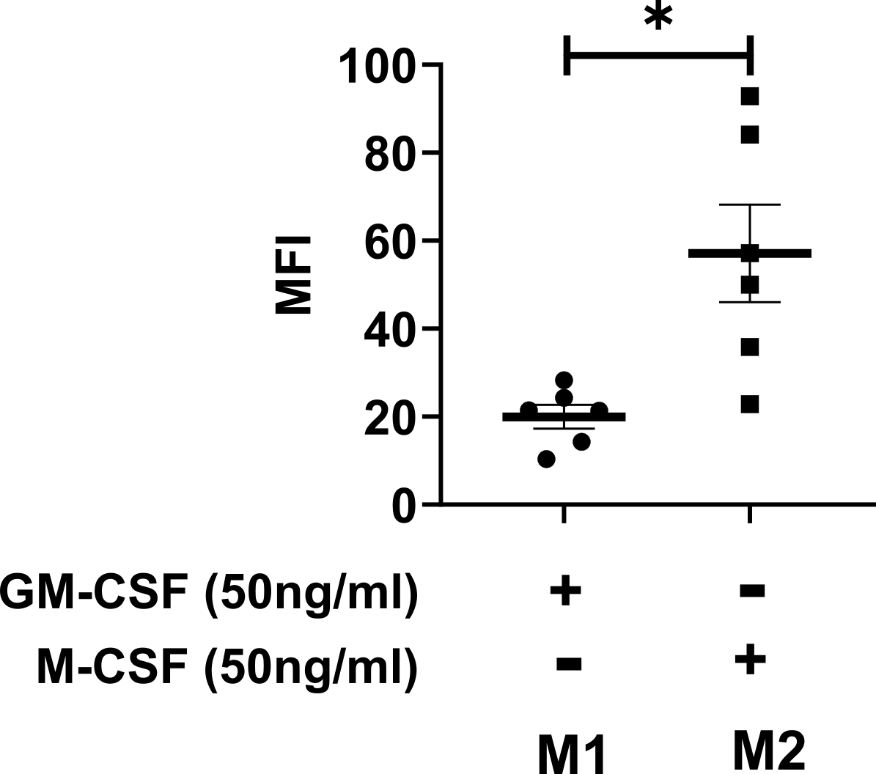
*

***Suppl Fig. 1: Surface Expression of CD163 on M1 and M2 macrophages.*** *Marker expression was determined by flow cytometry.* *Data are the mean values ± SEM (n=6). *p<0.05.*

*
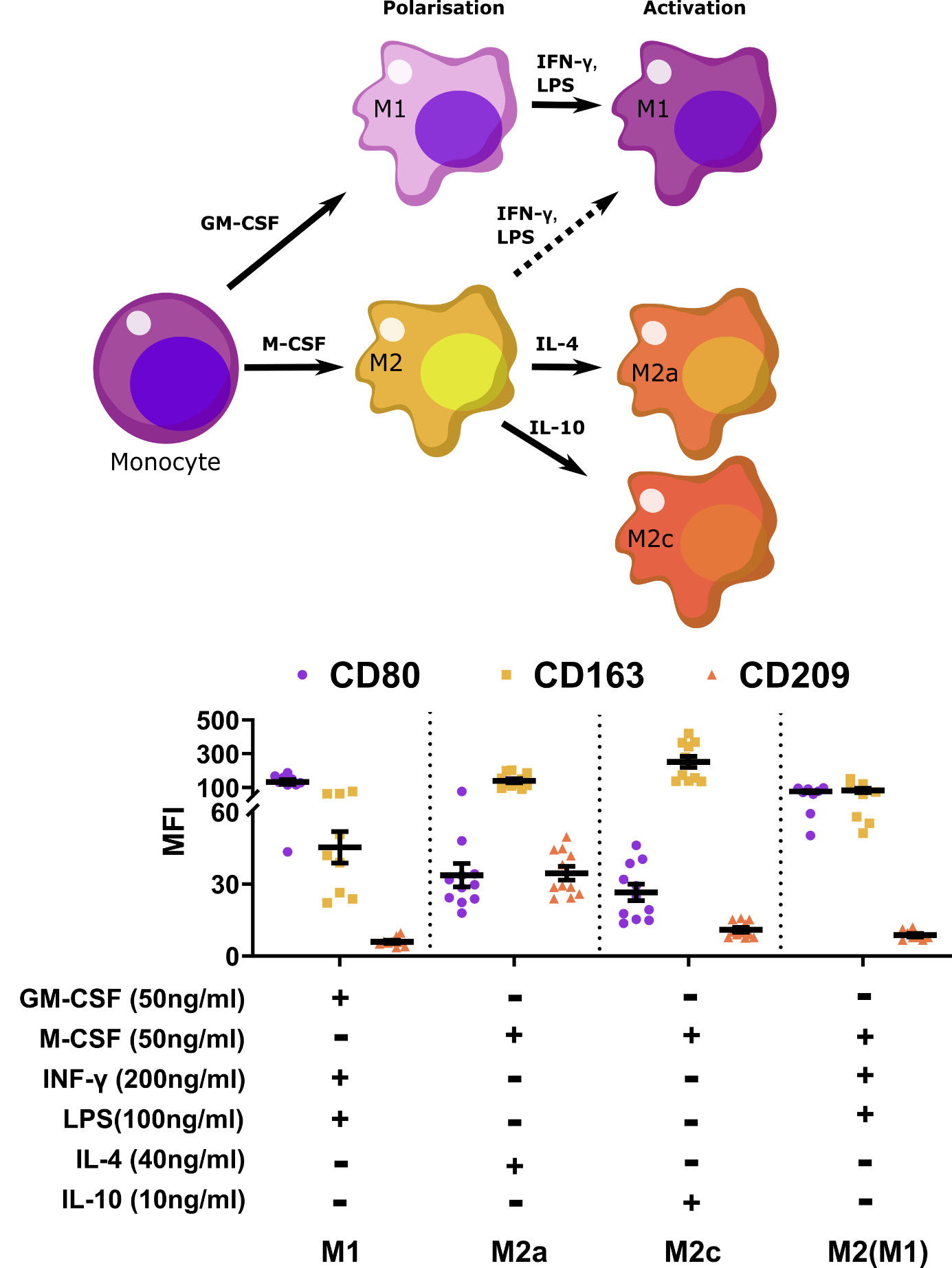
*

***Suppl Fig 2: Macrophage activation*** *Surface expression of CD80, CD163 and CD209 on activated macrophages was determined by flow cytometry.* *Data are the mean values ± SEM (n=9).*
